# The RIP and block-RIP analysis of Nyquist folding receiver for recovering signals

**DOI:** 10.1186/s13634-016-0392-5

**Published:** 2016-09-05

**Authors:** Kaili Jiang, Sujuan Chen, Bin Tang

**Affiliations:** School of Electronic Engineering, University of Electronic Science and Technology of China, Qingshuihe Campus: No. 2006, Xiyuan Ave, West Hi-Tech Zone, Chengdu, Sichuan China

**Keywords:** Nyquist folding receiver, Compressive sensing, Restricted isometry property, Block restricted isometry property, Signal reconstruction

## Abstract

Modern radar and communication systems require the detection and parameter estimation of signal under a broadband radio frequency (RF) environment. The Nyquist folding receiver (NYFR) is an efficient analog-to-information (A2I) architecture. It can use the compressive sensing (CS) techniques to break the limitations of the analog-to-digital converter (ADC). This paper demonstrates the restricted isometry property (RIP) of the NYFR deterministically by applying the Gershgorin circle theory. And, the NYFR suffers a poor RIP for the broadband signal, which will lead the conventional CS algorithms to be invalid. So, we derive the Fourier spectrum of the broadband signal, which covered multiple Nyquist zones and received by the NYFR. Then, the broadband signal can be regarded as the block-sparse signal. And, the block CS algorithms are applied for recovering the signal based on the analysis of the block-RIP. Finally, the simulation experiments demonstrate the validity of the findings.

## Introduction

Electronic warfare (EW) plays a leading role in most conflicts for future war, and the receiver is the core. The modern receivers require the detection and parameter estimation of signal across an extremely wide radio frequency (RF) bandwidth [1]. Meanwhile, the receiver is a trend to be much smaller on the size, weight, and power with a better performance. All of these set the urgent request to the higher performance of analog-to-digital converter (ADC), both in the sampling rate and analogy bandwidth [2].

The receiver, in general, is based on the Nyquist theorem [3] to avoid aliasing with redundancy. The channelized receivers use multichannel parallel alternating sampling in the time [4] or frequency [5] domain. The channelized receivers have a high intercept probability and sensitivity and wide instantaneous bandwidth and dynamic range. But, the multichannel structure is large in the size, weight, and power. With the development of the compressive sensing (CS) theory, the sparse signals can be recovered exactly by solving a convex optimization problem [6].

The Nyquist folding receiver (NYFR) is an efficient analog-to-information (A2I) architecture [7] which substantially preserves the signal structure [1]. Reference [1] discusses the CS framework of the NYFR and the signal reconstruction with single-frequency applying the orthogonal matching pursuit algorithm (OMP). References [8] and [9] regard the NYFR architecture as a modulated sampling scheme. They study the detection and parameter estimation algorithm of signal under the different types of the modulated signal.

On the other hand, the structure of the measurement matrix is a core problem in CS. The structured measurement matrix is constructed from the point of view of the practical application, such as the cyclic matrix [10], the Toeplitz matrix [11], and the Toeplitz-block matrices [12]. One of the main analysis tools of the measurement matrix is the restricted isometry property (RIP) [13]. And, reference [14] proves that the Toeplitz matrix satisfies the RIP. Reference [15] uses various techniques for demonstrating RIP deterministically including the Gershgorin circle theory. Besides, the block-sparse signals arise naturally, as the multiband signal [16], the radar imaging [17], and the DNA microarrays [18]. Then, reference [19] presents the block-RIP and the block CS algorithms as a block version of the orthogonal matching pursuit algorithm (BOMP).

In this paper, we demonstrate the RIP of the NYFR deterministically by applying the Gershgorin circle theory. And, we get the same conclusion as the reference [20] shown. That is, the NYFR suffers a poor RIP for the broadband signal, which will lead the conventional CS algorithms to be invalid. The broadband signal covers multiple Nyquist zones received by the NYFR, and then, we present its Fourier spectrum. And, the broadband signal can be regarded as the block-sparse signal. We demonstrate the block-RIP of the NYFR deterministically by using the property that the Toeplitz matrix satisfies the RIP. And finally, the simulation experiments give the broadband signal reconstruction by comparing the method of OMP and BOMP.

Then, the rest of this paper is organized as follows. Section 2 shows the CS model of the NYFR and analyzes the RIP by applying the Gershgorin circle theory. Section 3 presents the broadband signal model under the NYFR and demonstrates the block-RIP of the NYFR deterministically. Section 4 gives the simulation results and discussions. Section 5 concludes the whole paper.

## The RIP analysis of the Nyquist folding receiver

### Compressive sensing model of the NYFR

**Fig. 1:**
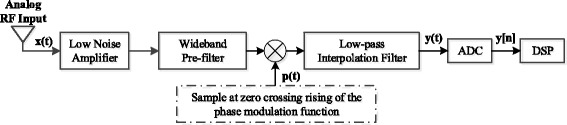
The Nyquist folding receiver architecture

The architecture of the NYFR is shown in Fig. 1, which is a twice sampling structure. The analog RF input can be subsampled by using a steam of short pulses *p*(*t*) that have a phase modulated sampling period as the first sampling. Followed by the low-pass interpolation filtering, the output is quantized by a conventional ADC as the second sampling.

The CS model of the NYFR based on the usual assumptions shown in reference [1] is

1 $$ y=\phi x\kern0.5em \mathrm{or}\kern0.5em y=\varPhi X $$

where *x* is the analog RF input vector sampled by using the Nyquist sampling rate in *C*^*N*^. The *N*-point discrete Fourier transform (DFT) vector *X* of *x* is sparse or called compressible in the frequency domain. The output of the NYFR is the vector *y* in *C*^*M*^. The matrix *ϕ* ∈ *C*^*M* × *N*^ is the sensing matrix, and *Φ* ∈ *C*^*M* × *N*^ is the measurement matrix. Note that *N* = *M* ⋅ *K*, where *K* denotes the total number of the Nyquist zones covered by the NYFR. So, we have

2 $$ \left[\begin{array}{c}\hfill {y}_1\hfill \\ {}\hfill {y}_2\hfill \\ {}\hfill \vdots \hfill \\ {}\hfill {y}_M\hfill \end{array}\right]=\underset{R}{\underbrace{\left[\begin{array}{cccc}\hfill {I}_M\hfill & \hfill {I}_M\hfill & \hfill \cdots \hfill & \hfill {I}_M\hfill \end{array}\right]}}\underset{S}{\underbrace{\left[\begin{array}{cccc}\hfill {I}_M\hfill & \hfill \hfill & \hfill \hfill & \hfill \hfill \\ {}\hfill \hfill & \hfill {e}^{-j\theta (t)}{I}_M\hfill & \hfill \hfill & \hfill \hfill \\ {}\hfill \hfill & \hfill \hfill & \hfill \ddots \hfill & \hfill \hfill \\ {}\hfill \hfill & \hfill \hfill & \hfill \hfill & \hfill {e}^{-j\left(K-1\right)\theta (t)}{I}_M\hfill \end{array}\right]}}\underset{\varPsi }{\underbrace{\left[\begin{array}{cccc}\hfill {\varPsi}_M\hfill & \hfill \hfill & \hfill \hfill & \hfill \hfill \\ {}\hfill \hfill & \hfill {\varPsi}_M\hfill & \hfill \hfill & \hfill \hfill \\ {}\hfill \hfill & \hfill \hfill & \hfill \ddots \hfill & \hfill \hfill \\ {}\hfill \hfill & \hfill \hfill & \hfill \hfill & \hfill {\varPsi}_M\hfill \end{array}\right]}}\left[\begin{array}{c}\hfill {X}_1\hfill \\ {}\hfill {X}_2\hfill \\ {}\hfill \vdots \hfill \\ {}\hfill {X}_M\hfill \\ {}\hfill \vdots \hfill \\ {}\hfill {X}_N\hfill \end{array}\right] $$

where each block of the block diagonal matrix *Ψ* ∈ *C*^*N* × *N*^ is an inverse DFT (IDFT) matrix *Ψ*_*M*_ ∈ *C*^*M* × *M*^ of size *M*, and each block represents a Nyquist zone. The induced sampling modulation matrix *S* ∈ *C*^*N* × *N*^ is a diagonal matrix with the function of time, whose modulation of phase is periodic nonuniform, and it is partitioned into blocks of size *M*. Then finally, each Nyquist zone is project into the baseband of bandwidth *M* with the matrix *R* ∈ *C*^*M* × *N*^. Note that the sensing matrix is *ϕ* = *RS* and the measurement matrix is *Φ* = *RSΨ*. The IDFT matrix of length *M* is given by

3 $$ {\varPsi}_M=\frac{1}{\sqrt{M}}\left[\begin{array}{ccccc}\hfill 1\hfill & \hfill 1\hfill & \hfill 1\hfill & \hfill \hfill & \hfill 1\hfill \\ {}\hfill 1\hfill & \hfill {\omega}^1\hfill & \hfill {\omega}^2\hfill & \hfill \cdots \hfill & \hfill {\omega}^{M-1}\hfill \\ {}\hfill 1\hfill & \hfill {\omega}^2\hfill & \hfill {\omega}^4\hfill & \hfill \cdots \hfill & \hfill {\omega}^{2\left(M-1\right)}\hfill \\ {}\hfill \cdots \hfill & \hfill \cdots \hfill & \hfill \cdots \hfill & \hfill \cdots \hfill & \hfill \cdots \hfill \\ {}\hfill 1\hfill & \hfill {\omega}^{M-1}\hfill & \hfill {\omega}^{2\left(M-1\right)}\hfill & \hfill \cdots \hfill & \hfill {\omega}^{\left(M-1\right)\left(M-1\right)}\hfill \end{array}\right] $$

where *ω* = *e*^*j*2*π*/*M*^ is a rotation factor.

### The RIP analysis of the NYFR

As we know, the measurement matrix Φ satisfies the RIP, which is a sufficient condition for sparse reconstruction. The RIP is defined as:

A matrix *Φ* ∈ *C*^*M* × *N*^ is said to satisfy the RIP with parameters (*s*, *δ*) for *s* ≤ *M*, 0 ≤ *δ* ≤ 1, if for all subsequence index sets *I* ⊂ {1, 2, …, *N*} of Φ such that |*I*| ≤ *s*, and for all *θ* ∈ *C*^|*I*|^, one has

4 $$ \left(1-\delta \right){\left\Vert \theta \right\Vert}_2^2\le {\left\Vert {\varPhi}_I\theta \right\Vert}_2^2\le \left(1+\delta \right){\left\Vert \theta \right\Vert}_2^2 $$

where | ⋅ | is the cardinality of the set, which means the number of the set of elements. And, the infimum of all *δ* is the restricted isometry constant (RIC) *δ*_*s*_. There is a relation of inequality between the RIC and the eigenvalues of the matrix ${\varPhi}_I^H{\varPhi}_I$ [21]

5 $$ 1-{\delta}_s\le {\lambda}_{\min}\left({\varPhi}_I^H{\varPhi}_I\right)\le {\lambda}_{\max}\left({\varPhi}_I^H{\varPhi}_I\right)\le 1+{\delta}_s $$

where ${\lambda}_{\min}\left({\varPhi}_I^H{\varPhi}_I\right)$ and ${\lambda}_{\max}\left({\varPhi}_I^H{\varPhi}_I\right)$ denote the minimal and maximal eigenvalues of ${\varPhi}_I^H{\varPhi}_I$, respectively.

Then, we can apply Gershgorin circle theorem [22] to understand the RIP. Considering the measurement matrix *Φ* = *RSΨ*, there are ${C}_N^s$ submatrices *Φ*_*I*_ on a random selection of *s* columns. So, the eigenvalues of each submatrix of Gram ${\varPhi}_I^H{\varPhi}_I$ distributed in [1 − *δ*_*s*_, 1 + *δ*_*s*_] are a complicated permutation and combination problem. According to Gershgorin circle theorem, the Gram matrix of Φ contains all the eigenvalue information of its submatrices. So, it is reasonable to analyze the Gram matrix *G*(Φ), which is given by

6 $$ G\left(\varPhi \right)={\varPhi}^H\varPhi ={\varPsi}^H\left({S}^H\left({R}^HR\right)S\right)\varPsi =\left[\begin{array}{cccc}\hfill {I}_M\hfill & \hfill {T}_{10}\hfill & \hfill \cdots \hfill & \hfill {T}_{\left(K-1\right)0}\hfill \\ {}\hfill {T}_{01}\hfill & \hfill {I}_M\hfill & \hfill \cdots \hfill & \hfill {T}_{\left(K-1\right)1}\hfill \\ {}\hfill \cdots \hfill & \hfill \cdots \hfill & \hfill \cdots \hfill & \hfill \cdots \hfill \\ {}\hfill {T}_{0\left(K-1\right)}\hfill & \hfill {T}_{1\left(K-1\right)}\hfill & \hfill \cdots \hfill & \hfill {I}_M\hfill \end{array}\right] $$

where *I*_*M*_ is an identity matrix of size *M* × *M*. And, the square matrix *T*_*ij*_ (*i*, *j* = 0, 1, ⋯ *K* − 1; *i* ≠ *j*) of size *K* × *K* is expressed as

7 $$ {T}_{ij}=\frac{1}{M}\left[\begin{array}{cccc}\hfill {\displaystyle \sum_{m=1}^M{e}^{-j{k}_{ij}\theta \left({t}_m\right)}}\hfill & \hfill {\displaystyle \sum_{m=1}^M{e}^{-j{k}_{ij}\theta \left({t}_m\right)}}{\omega}^{\left(m-1\right)}\hfill & \hfill \cdots \hfill & \hfill {\displaystyle \sum_{m=1}^M{e}^{-j{k}_{ij}\theta \left({t}_m\right)}}{\omega}^{\left(m-1\right)\left(M-1\right)}\hfill \\ {}\hfill {\displaystyle \sum_{m=1}^M{e}^{-j{k}_{ij}\theta \left({t}_m\right)}}{\omega}^{-\left(m-1\right)}\hfill & \hfill {\displaystyle \sum_{m=1}^M{e}^{-j{k}_{ij}\theta \left({t}_m\right)}}\hfill & \hfill \cdots \hfill & \hfill {\displaystyle \sum_{m=1}^M{e}^{-j{k}_{ij}\theta \left({t}_m\right)}}{\omega^{\left(m-1\right)}}^{\left(M-2\right)}\hfill \\ {}\hfill \cdots \hfill & \hfill \cdots \hfill & \hfill \cdots \hfill & \hfill \cdots \hfill \\ {}\hfill {\displaystyle \sum_{m=1}^M{e}^{-j{k}_{ij}\theta \left({t}_m\right)}}{\omega^{-\left(m-1\right)}}^{\left(M-1\right)}\hfill & \hfill {\displaystyle \sum_{m=1}^M{e}^{-j{k}_{ij}\theta \left({t}_m\right)}}{\omega^{-\left(m-1\right)}}^{\left(M-2\right)}\hfill & \hfill \hfill & \hfill {\displaystyle \sum_{m=1}^M{e}^{-j{k}_{ij}\theta \left({t}_m\right)}}\hfill \end{array}\right] $$

where *k*_*ij*_ = *k*_*i*_ − *k*_*j*_, the *k*_*i*_ means *i*th-index Nyquist zone and *k*_*j*_ is the same. To simplify the matrix (7), we have

8 $$ {T}_{ij}={\left(\frac{1}{M}{\displaystyle \sum_{m=1}^M{e}^{-j{k}_{ij}\theta \left({t}_m\right)}}{\omega^{\left(m-1\right)}}^{\left(l-n\right)}\right)}_{l,n}\left(l,n=1,2\cdots M\right) $$

where *n* and *l* denote the row and column of the matrix *T*_*ij*_, respectively.

We begin our analysis with the 1-sparse. Because the diagonal elements of the Gram matrix are equal to 1 identically, which means that Φ meets 1-RIP, the signal can be recovered exactly when the input is 1-sparse. Moreover, considering 2-sparse, we choose *δ*_*d*_, *δ*_0_ > 0 appropriately and *δ*_*d*_ + *δ*_0_ = *δ*_*s*_ ∈ (0, 1), to be made that the diagonal elements *G*_*i*,*i*_ of the Gram matrix satisfy |*G*_*i*,*i*_ − 1| = 0 < *δ*_*d*_ and the off-diagonal elements meet the relationship |*G*_*i*,*j*_ − 1| < *δ*_*o*_/*s*. In other words, the distance between the center of Gershgorin circle and 1 is no further than *δ*_*d*_, and the radius of each Gershgorin circle is less than *δ*_0_. Then, we can set *δ*_*d*_ to an extremely tiny positive number, and we have *δ*_*o*_ ≈ *δ*_*s*_. So, what we need to do is that proving each of the non-diagonal elements from the matrix *G*(Φ) is less than 1.

The elements of the matrix *T*_*ij*_ can be written as

9 $$ {b}_{nl}=\frac{1}{M}{\displaystyle \sum_{m=1}^M{e}^{-j{k}_{ij}\theta \left({t}_m\right)}}{\omega^{\left(m-1\right)}}^{\left(l-n\right)} $$

and

10 $$ \left|{b}_{nl}\right|=\left|\frac{1}{M}{\displaystyle \sum_{m=1}^M{e}^{-j{k}_{ij}\theta \left({t}_m\right)}}{\omega^{\left(m-1\right)}}^{\left(l-n\right)}\right|\le \frac{1}{M}\left(\left|{e}^{-j{k}_{ij}\theta \left({t}_1\right)}\right|+\left|{e}^{-j{k}_{ij}\theta \left({t}_2\right)}{\omega}^{\left(l-n\right)}\right|+\cdots \right.+\left.\left|{e}^{-j{k}_{ij}\theta \left({t}_K\right)}{\omega^{\left(M-1\right)}}^{\left(l-n\right)}\right|\right)=1 $$

where *b*_*nl*_ is equal to 1 if and only if *θ*(*t*_*m*_) = 0. However, *θ*(*t*) is not a fixed value; instead, it is a function.

After that

11 $$ \left|{b}_{nl}\right|<1 $$

So, the eigenvalues of the matrix which is composed of arbitrary two columns of Φ are between 0 and 1, namely that the measurement matrix of the NYFR satisfies 2-RIP. The result is the same as that OMP will recover any two tones from their samples regardless of any disparity on the reference [20]. However, equation (11) also shows that the sensing matrix of the NYFR not satisfies 3-RIP or more, because there is a case that the eigenvalues of the matrix which is composed of arbitrary more than two columns of Φ may be over one.

The RIP is a useful tool in the analysis of the measurement matrix of the CS methods, even if the estimation of the NYFR suffers a good RIP as is shown in reference [20], which will not guarantee the success of the CS algorithms in discriminating the broadband signal. So, the conversion of broadband signal based on the CS is a challenge, and the block-RIP will offer some improvement.

## The block-RIP analysis of the Nyquist folding receiver

### Broadband signal model under the NYFR

To make the description easier, we use a broadband linear frequency modulation (LFM) signal which covered multiple Nyquist zones as an example. And, the Fourier spectrum of the broadband signal is not satisfied sparse condition. Assuming that the signal amplitude is *A*_*c*_, the pulse width is *τ*, the initial frequency is *ω*_*c*_, the initial phase is *φ*_*c*_ and the chirp rate is *μ*, then the form of the broadband signal can be expressed as

12 $$ x(t)={A}_c\mathrm{rect}\left(\frac{t}{\tau}\right) \exp \left[j\left({\omega}_ct+\pi \mu {t}^2+{\varphi}_c\right)\right] $$

where rect(u) is the rectangle function as follow

13 $$ \mathrm{rect}(u)=\left\{\begin{array}{c}\hfill 1\kern1em \left|u\right|\le 0.5\hfill \\ {}\hfill 0\kern1em \left|u\right|\ge 0.5\hfill \end{array}\right. $$

The Fourier spectrum of LFM can be expressed as

14 $$ X\left(\omega \right)=\left\{\begin{array}{cc}\hfill {A}_c\sqrt{\frac{2\pi }{\mu }} \exp \left(-j\left(\frac{{\left(\omega -{\omega}_c\right)}^2}{2\mu }-\frac{\pi }{4}\right)+j{\varphi}_c\right)\hfill & \hfill \left|\omega -{\omega}_c\right|\le \frac{\triangle \omega }{2}\hfill \\ {}\hfill 0\hfill & \hfill \left|\omega -{\omega}_c\right|\ge \frac{\triangle \omega }{2}\hfill \end{array}\right. $$

where *Δω* = 2*πB*, and *B* = *μτ* is the bandwidth.

And, the RF sample clock based on the NYFR is described in reference [1] as follow:

15 $$ p(t)={p}_{\mathrm{model}}(t)\ast {\omega}_s{\displaystyle \sum_{k=0}^K{e}^{jk\left[{\omega}_st+\theta (t)\right]}} $$

where *p*_model_(*t*) is the impulse of the model. And *k* represents the index of Nyquist zones from zero to *K*, where *K* denotes the number of the Nyquist zones by the NYFR covered. From the signal modulation theory perspective, ${\omega}_s{\displaystyle \sum_{k=0}^K{e}^{jk\left[{\omega}_st+\theta (t)\right]}}$ can modulate the Nyquist zone information of the input into the bandwidth information of the received signal. Then, the expression can be simplified to

16 $$ p(t)={\omega}_s{\displaystyle \sum_{k=0}^K{e}^{jk\left[{\omega}_st+\theta (t)\right]}} $$

where assuming *θ*(*t*) = sin(*ω*_*θ*_*t*) is the sinusoid function for phase modulated.

Using the Jacobi identity

17 $$ \exp \left(j\alpha \sin \beta \right)={\displaystyle \sum_{\nu =-\infty}^{+\infty }{J}_{\nu}\left(\alpha \right)} \exp \left(j\nu \beta \right) $$

And, the sinusoid phase modulated function, we have

18 $$ p(t)={\omega}_s{\displaystyle \sum_{k=0}^K{\displaystyle \sum_{\nu =-\infty}^{+\infty }{J}_{\nu }(k)} \exp \left(jk{\omega}_st+j2\pi {f}_{\theta}\nu t\right)} $$

and

19 $$ P\left(\omega \right)={\omega}_s{\displaystyle \sum_{k=0}^K{\displaystyle \sum_{\nu =-\infty}^{+\infty }{J}_{\nu }(k)}\delta \left(\omega -k{\omega}_s-\nu {\omega}_{\theta}\right)} $$

Then, we have

20 $$ \begin{array}{l}{Y}^{\prime}\left(\omega \right)=\frac{1}{2\pi }X\left(\omega \right)\ast P\left(\omega \right)\\ {}\kern4.5em =\frac{A_c{\omega}_s}{\sqrt{2\pi \mu }}{\operatorname{e}}^{j\left({\varphi}_c+\frac{\pi }{4}\right)}{\displaystyle \sum_{v=-\infty}^{\infty }{\displaystyle \sum_{k=0}^K{J}_v(k)} \exp \left(-j\frac{{\left(\omega -{\omega}_c-k{\omega}_s-v{\omega}_{\theta}\right)}^2}{2\mu}\right)}\\ {}\kern4.5em =\frac{A_c{\omega}_s}{2\pi }{\operatorname{e}}^{j{\varphi}_c}{\displaystyle \sum_{v=-\infty}^{\infty }{\displaystyle \sum_{k=0}^K{J}_v(k)}\left(\sqrt{\frac{2\pi }{\mu }} \exp \left(-j\left(\frac{{\left(\omega -\left({\omega}_c+k{\omega}_s+v{\omega}_{\theta}\right)\right)}^2}{2\mu }-\frac{\pi }{4}\right)\right)\right)}\end{array} $$

Then, we can see that *ω*_*c*_ + *kω*_*s*_ is the mid-frequency with the edge frequencies separated by the amount of *ω*_*θ*_ and the amplitudes depend on Bessel.

Through a low-pass interpolation filter of [‐ *ω*_*s*_/2, *ω*_*s*_/2], the spectrum can be represented as:

21 $$ F\left(\omega \right)=\left\{\begin{array}{cc}\hfill 1,\hfill & \hfill 0\le \left|\omega \right|\le {\omega}_s/2\hfill \\ {}\hfill 0,\hfill & \hfill {\omega}_s/2<\left|\omega \right|\le 2\pi \hfill \end{array}\right. $$

Then, the range of the Nyquist zones of the input is determined by *F(ω)* as follow

22 $$ \left\{\begin{array}{c}\hfill -{\omega}_s/2\le {\omega}_c+{\omega}_s{k}_{\mathrm{up}}\le {\omega}_s/2\hfill \\ {}\hfill -{\omega}_s/2\le {\omega}_c+2\pi B+{\omega}_s{k}_{\mathrm{down}}\le {\omega}_s/2\hfill \end{array}\right.,\kern1.5em k\in Z $$

where *k*_up_ and *k*_down_ denote the minimal and maximal index values of the Nyquist zones of the input, respectively.

Because the instantaneous frequency of the LFM signal is linear with time, so we have

23 $$ {k}_1={k}_{\mathrm{up}},{k}_2={k}_{\mathrm{up}}-1,\kern0.5em \dots, \kern0.5em {k}_{\varDelta -1}={k}_{\mathrm{down}}+1,\kern0.5em {k}_{\varDelta }={k}_{\mathrm{down}} $$

And, the bandwidth values correspondingly in the different Nyquist zones are

24 $$ \begin{array}{l}{B}_1=\left(1/2-{k}_{\mathrm{up}}\right){\omega}_s-{\omega}_c\\ {}{B}_2={B}_3=\cdots ={B}_{\varDelta -1}={\omega}_s\\ {}{B}_{\varDelta }=\left(1/2+{k}_{\mathrm{down}}\right){\omega}_s\kern0.5em +{\omega}_c+2\pi B\kern0.5em \end{array} $$

Without change of the chirp rate, then the pulse width values of different Nyquist zones are

25 $$ {\tau}_i={B}_i/\mu $$

where *i* = 1, 2, …, *Δ*. Then, the initial frequencies in the different Nyquist zones are ${\omega}_c+{k}_i{\omega}_s+{\displaystyle \sum_{l=0}^{i-1}{B}_l}$, respectively.

The output spectrum of the NYFR after the low-pass interpolation filter is

26 $$ \begin{array}{l}Y\left(\omega \right)={Y}^{\prime}\left(\omega \right)F\left(\omega \right)\\ {}\kern4em =\frac{A_c{\omega}_s}{2\pi }{\operatorname{e}}^{j{\varphi}_c}{\displaystyle \sum_{v=-\infty}^{\infty }{\displaystyle \sum_{i=1}^{\varDelta }{J}_v\left({k}_i\right)\left(\sqrt{\frac{2\pi }{\mu }} \exp \left(-j\left(\frac{{\left(\omega -\left({\omega}_c+{k}_i{\omega}_s+{\displaystyle \sum_{l=0}^{i-1}{B}_l}\kern0.5em +v{\omega}_{\theta}\right)\right)}^2}{2\mu }-\frac{\pi }{4}\right)\right)\right)}}\\ {}\kern4.5em \\ {}\kern4em \end{array} $$

where assuming *B*_0_ = 0.

With the Fourier transform pair,

27 $$ {e}^{j2\pi a{t}^2}={\left.{e}^{-2\pi \alpha {t}^2}\right|}_{\alpha =-ja,\kern0.5em a=j\alpha}\overset{\Im }{\leftrightarrow}\sqrt{\frac{\pi }{a}} \exp \left(-j\left(\frac{\pi^2{f}^2}{a}-\frac{\pi }{4}\right)\right) $$

where *a* = *μ*/2, then $$ \alpha =-j\left(\mu /2\right),\kern0.5em \sqrt{\pi /a}=\sqrt{2\pi /\mu } $$.

Then, we have

28 $$ \begin{array}{l}y(t)=\frac{A_c{\omega}_s}{2\pi }{\displaystyle \sum_{v=-\infty}^{\infty }{\displaystyle \sum_{i=1}^{\varDelta }{J}_v\left({k}_i\right)\mathrm{rect}\left(\frac{t}{\tau_i}\right){e}^{j\left({\omega}_c+{k}_i{\omega}_s+{\displaystyle \sum_{l=0}^{i-1}{B}_l}+v{\omega}_{\theta}\right)t}{e}^{j\pi \mu {t}^2}{\operatorname{e}}^{j{\varphi}_c}}}\\ {}\kern3.5em =\frac{A_c{\omega}_s}{2\pi }{\displaystyle \sum_{i=1}^{\varDelta}\mathrm{rect}\left(\frac{t}{\tau_i}\right) \exp \left(j\left(\left({\omega}_c+{k}_i{\omega}_s+{\displaystyle \sum_{l=0}^{i-1}{B}_l}\right)t+\pi \mu {t}^2+{\varphi}_c+{k}_i \sin \left({\omega}_{\theta }t\right)\right)\right)}\end{array} $$

So, the broadband signal received by the NYFR often has the aliasing spectrum from subsampling, especially for the signal of the high frequency, broad bandwidth and covering multiple Nyquist zones. Therefore, the traditional RIP analysis and CS methods will not work. And, the block-RIP analysis and block CS methods will offer some improvement.

### The block-RIP analysis of the NYFR

We consider that the *N*-point vector *X* can be divided into *L* blocks on the sub-block index sets *D* = {*d*_1_, *d*_2_, …, *d*_*L*_}. The length of each block is *d* fixation, and there is *dL = N*. Denoting that *X*[*l*] is the *l*th sub-block of length *d*, we can rewrite *X* as

29 $$ {X}^T=\left[\underset{X\left[1\right]}{\underbrace{X_1\cdots {X}_{d_1}}}\cdots \underset{X\left[l\right]}{\underbrace{X_{d_{l-1}+1}\cdots {X}_{d_l}}}\cdots \underset{X\left[L\right]}{\underbrace{X_{N-{d}_L+1}\cdots {X}_N}}\right] $$

Similarly, we can represent Φ as a concatenation of column-block Φ[*l*] of size M × d

30 $$ \varPhi =\left[\underset{\varPhi \left[1\right]}{\underbrace{\varphi_1\cdots {\varphi}_d}}\cdots \underset{\varPhi \left[l\right]}{\underbrace{\varphi_{d_{l-1}+1}\cdots {\varphi}_{d_l}}}\cdots \underset{\varPhi \left[L\right]}{\underbrace{\varphi_{N-{d}_L+1}\cdots {\varphi}_N}}\right] $$

Then, Φ has the block-RIP over *D* with parameter *δ*_*B*_ ∈ [0, 1] if for all *θ* ∈ *C*^*N*^ which is block *s*-sparse over *D*, we have that

31 $$ \left(1-{\delta}_B\right){\left\Vert \theta \right\Vert}_2^2\le {\left\Vert \varPhi \theta \right\Vert}_2^2\le \left(1+{\delta}_B\right){\left\Vert \theta \right\Vert}_2^2 $$

The infimum of all *δ*_*B*_ is the block restricted isometry constant (block-RIC) *δ*_*s*|*D*_. We can see that the RIP is for all the subsequence index sets of Φ, while the block-RIP is for the measurement matrix Φ itself. Compared with the inequality of RIC.

32 $$ {\lambda}_{\min}\left({\varPhi}_I^H{\varPhi}_I\right){\left\Vert \theta \right\Vert}_2^2\le {\left\Vert {\varPhi}_I\theta \right\Vert}_2^2={\theta}^H{\varPhi_I}^H{\varPhi}_I\theta \le {\lambda}_{\max}\left({\varPhi}_I^H{\varPhi}_I\right){\left\Vert \theta \right\Vert}_2^2 $$

And, we can let ${\delta}_s= \max \left\{1-{\lambda}_{\min}\left({\varPhi}_I^H{\varPhi}_I\right),1+{\lambda}_{\max}\left({\varPhi}_I^H{\varPhi}_I\right)\right\}$. Correspondingly, the inequality of the block-RIC with the parameter of the sub-coherence *υ* and the block-coherence *μ*_*B*_ shown in [19] is changed into

33 $$ \begin{array}{c}{\left\Vert \varPhi \theta \right\Vert}_2^2={\theta}^H{\varPhi}^H\varPhi \theta ={\displaystyle \sum_{c=1}^s{\displaystyle \sum_{r=1}^s{\theta}^H\left[c\right]M\left[c,r\right]\theta \left[r\right]}}\\ {}\kern0.5em ={\displaystyle \sum_{c=1}^s{\theta}^H\left[c\right]M\left[c,c\right]\theta \left[c\right]}+{\displaystyle \sum_{c=1}^s{\displaystyle \sum_{r=1,r\ne c}^s{\theta}^H\left[c\right]M\left[c,r\right]\theta \left[r\right]}}\\ {}\kern0.5em \le {\left\Vert \theta \right\Vert}_2^2+{\displaystyle \sum_{c=1}^s max\rho \left(M\left[c,c\right]-{\mathbf{I}}_d\right)}{\left\Vert \theta \right\Vert}_2^2+{\displaystyle \sum_{c=1}^s{\displaystyle \sum_{r=1,r\ne c}^s max\rho \left(M\left[c,r\right]\right){\theta}^H\left[c\right]\theta \left[r\right]}}\\ {}\kern0.5em \le \left[1+\left(d-1\right)\upsilon +\left(s-1\right)d{\mu}_B\right]{\left\Vert \theta \right\Vert}_2^2\end{array} $$

and

34 $$ \begin{array}{c}{\left\Vert \Phi \theta \right\Vert}_2^2={\displaystyle \sum_{c=1}^s{\theta}^H\left[c\right]M\left[c,c\right]\theta \left[c\right]}+{\displaystyle \sum_{c=1}^s{\displaystyle \sum_{r=1,r\ne c}^s{\theta}^H\left[c\right]M\left[c,r\right]\theta \left[r\right]}}\\ {}\kern0.5em \ge {\left\Vert \theta \right\Vert}_2^2-{\displaystyle \sum_{c=1}^s max\rho \left(M\left[c,c\right]-{\mathbf{I}}_d\right)}{\left\Vert \theta \right\Vert}_2^2-{\displaystyle \sum_{c=1}^s{\displaystyle \sum_{r=1,r\ne c}^s max\rho \left(M\left[c,r\right]\right){\theta}^H\left[c\right]\theta \left[r\right]}}\\ {}\kern0.5em \ge \left[1-\left(d-1\right)\upsilon -\left(s-1\right)d{\mu}_B\right]{\left\Vert \theta \right\Vert}_2^2\end{array} $$

where *M*[*c*, *r*] = *Φ*^*H*^[*c*]*Φ*[*r*], and *ρ*(⋅) is the singular value of the matrix. Then, we can let that

35 $$ {\delta}_{s\left|D\right.}=\left(d-1\right)\upsilon +\left(s-1\right)d{\mu}_B $$

To emphasize the advantage and the efficiency of the block-RIP analysis, we consider a special case of the NYFR. The measurement matrix is separated into *K* blocks of *M* columns each, namely *L = K*. In this example, the blocking is based on the unit of Nyquist zone, and the sub-coherence parameter of each block equals to zero approximately. Note that, for any input of block 1-sparse, which corresponds to *M* continuous non-zero values, the block-RIP is satisfied with *δ*_1|*D*_ ≈ 0. However, the RIP analysis shows that the measurement matrix of the NYFR satisfies 2-RIP of the maximal, which corresponds to at most two non-zero elements.

And, increasing the number of non-zero values to the block 2-sparse, we should calculate the block-coherence parameter *μ*_*B*_, and starting with the property of the matrix *M*. From the RIP analysis of the NYFR, we have

36 $$ M\left[c,r\right]={T}_{\left(c-1\right)\left(r-1\right)} $$

where *c*, *r* = 1, 2, ⋯ *K*; *c* ≠ *r*. We know that the matrix *T*_(*c* − 1)(*r* − 1)_ is a Toeplitz matrix from the equation (7). And because the Toeplitz matrix satisfies the RIP as the reference [14], there is ${\lambda}_{\max}^{1/2}\left({T}_{\left(c-1\right)\left(r-1\right)}^H{T}_{\left(c-1\right)\left(r-1\right)}\right)\in \left[0,1\right]$. Then, we have *dμ*_*B*_ ∈ [0, 1] and *δ*_2|*D*_ ≈ 1 which is satisfied by the lower bound of the block-RIP. Consequently, we can use the block CS algorithms to specify the input uniquely when the block sparse is no more than two, especially for the broadband signal receiving.

## Simulation results and discussions

In this section, we will verify the correctness of the RIP and block-RIP theoretical findings of the NYFR through the simulation examples. And, we will discuss about the sparse/block-sparse signal reconstruction under the analysis of the RIP and block-RIP. The simulation settings are specified in the Table 1 below.

**Table 1:** The simulation settings table of the NYFR

Average sampling frequency	*f*_*s*_	1 GHz
Sinusoid modulation frequency	*f*_*θ*_	1 MHz
The total number of Nyquist zones	*K*	4
The frequency of ADC	*f*_*ad*_	2 GHz
Simulation duration	*tao*	0.1 μs

### Scenario I: the simulation analysis verified the RIP and block-RIP theoretical findings of the NYFR

**Fig. 2:**
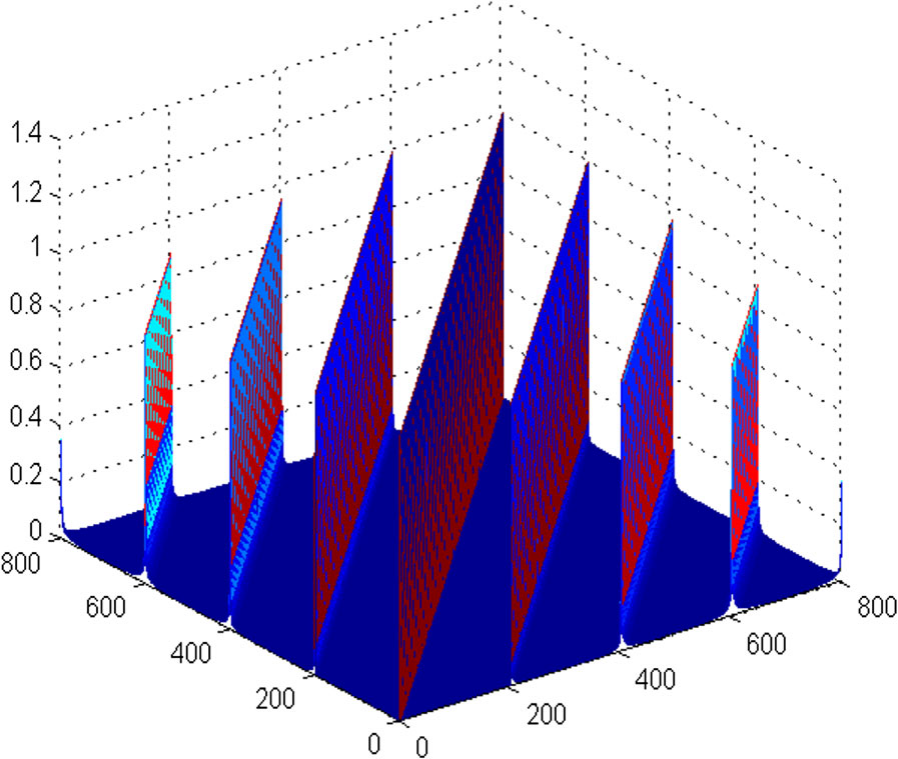
The elemental maps of the Gram matrix ${\varPhi}_I^H{\varPhi}_I$

We use sinusoid phase modulation here and have *M* = 200, *N* = *M* ⋅ *K* = 800. Figure 2 shows the elemental maps of the Gram matrix ${\Phi}_I^H{\Phi}_I$ of the measurement matrix of the NYFR. It indicates that the main diagonal elements are one, and the amplitudes of the non-diagonal elements are limited in [0,1]. So, the measurement matrix of the NYFR did not satisfy 3-RIP or more.

On the other hand, the majority of the non-zero amplitudes of the non-diagonal elements are located at the diagonal elements by the *M* = 200 intervals, except the main diagonal elements. Therefore, we will discuss the block-RIP of the special block case, which is partitioned into blocks with the unit of Nyquist zone.

**Fig. 3:**
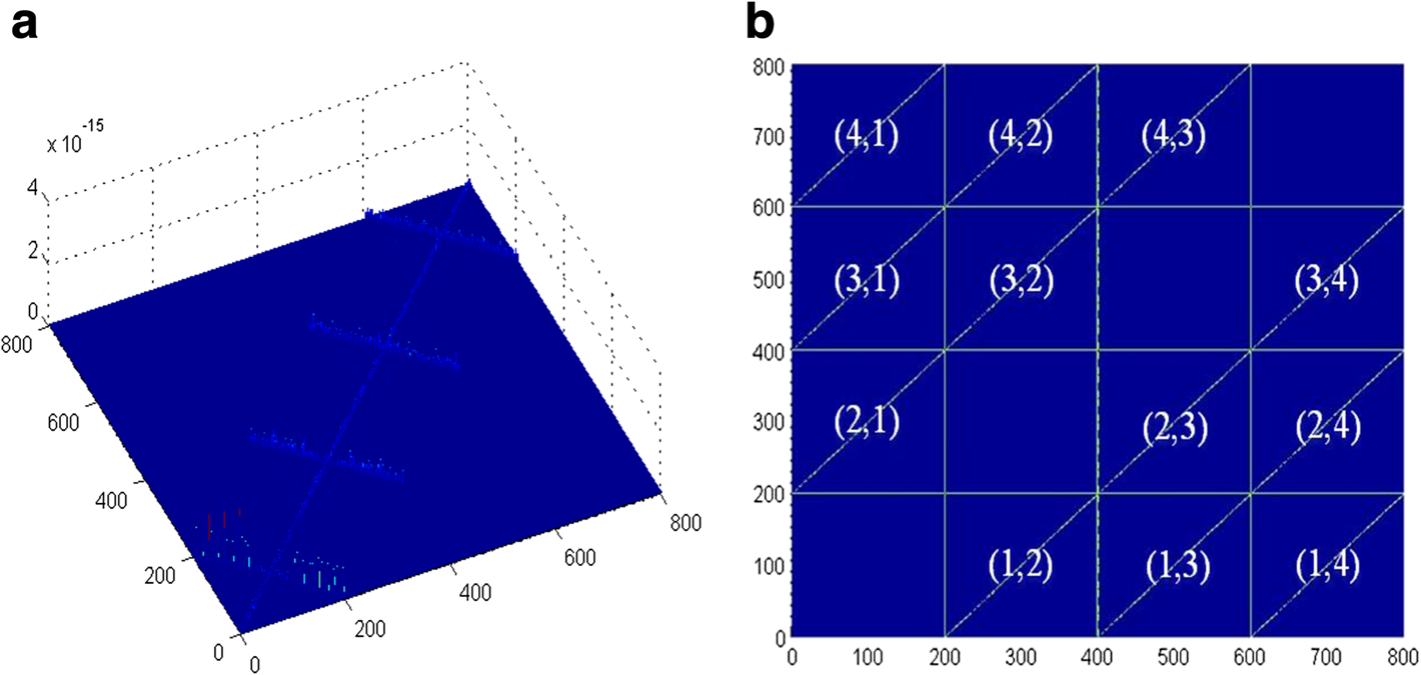
The elemental maps of the sub-coherence *υ* and the block-coherence *μ*_*B*_

As Fig. 3a shows, the elements of the sub-coherence *υ* are essentially zero, which means that there is the effective block CS algorithm to specify any the input of block 1-sparse uniquely. And, Fig. 3b is the elemental map of the Gram matrix ${T}_{\left(c-1\right)\left(r-1\right)}^H{T}_{\left(c-1\right)\left(r-1\right)}$, and it shows that the main diagonal elements of each blocks are one and the amplitudes of the non-diagonal elements are essentially zero. That verifies *dμ*_*B*_ ∈ [0, 1] and *δ*_2|*D*_ ≈ 1 which is satisfied by the lower bound of the block-RIP.

### Scenario II: the sparse/block-sparse signal reconstruction under the RIP and block-RIP analysis

**Fig. 4:**
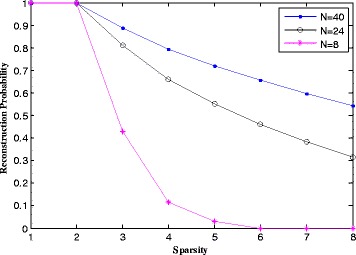
The relationship between reconstruction probability and sparsity of the RIP

Figure 4 is the relationship between reconstruction probability and sparsity of the RIP. To save the computer time and storage in the simulation, the sparsity sets to 1–8. And, the simulation duration is 1, 3, and 5 ns, that corresponds to *N* = 8, 24, 40. The reconstruction probability is calculated using 100 Monte Carlo trials for each duration value. And, we can see that the reconstruction probability is 100 % when the sparsity is 1 or 2, regardless of the points’ value. That is, the RIC is an absolute value, and the block-RIC is the same. The lower bound of the RIP/block-RIP is identified only by the architecture of the system and will not change with the parameters of the system. In other words, this also verifies that the RIP/block-RIP is a sufficient but not necessary condition. So, we cannot use the RIP/block-RIP to design the suitable observation matrix or receiver. However, the reconstruction probability increases with the sampling points increase in RIP-less.

As we know, block-RIP is the RIP, when the block size and interval are one. And, we can get the same conclusion that the reconstruction probability is 100 % when the sparsity is 1 or 2 as shown in the first row of the Table 2. For another, it is difficult to calculate the block-RIP in different blocks, because there is too many partition blocks. So, the adaptive recognition algorithm of the partition blocks based on the CS is worth studying.

**Table 2:** The relationship between the parameters of the block-RIP

Block size	Block interval	Sub-coherence	Block-coherence	*δ*_2|*D*_
1	1	0	0.9855	0.9855
2	1	0.0853	0.5318	>1
2	2	0	0.4994	0.4994
5	5	0	0.2	1
10	10	0	0.1	1
20	20	0	0.05	1
40	40	0	0.025	1
50	50	0	0.02	1
100	100	0	0.01	1
200	200	0	0.005	1

**Fig. 5:**
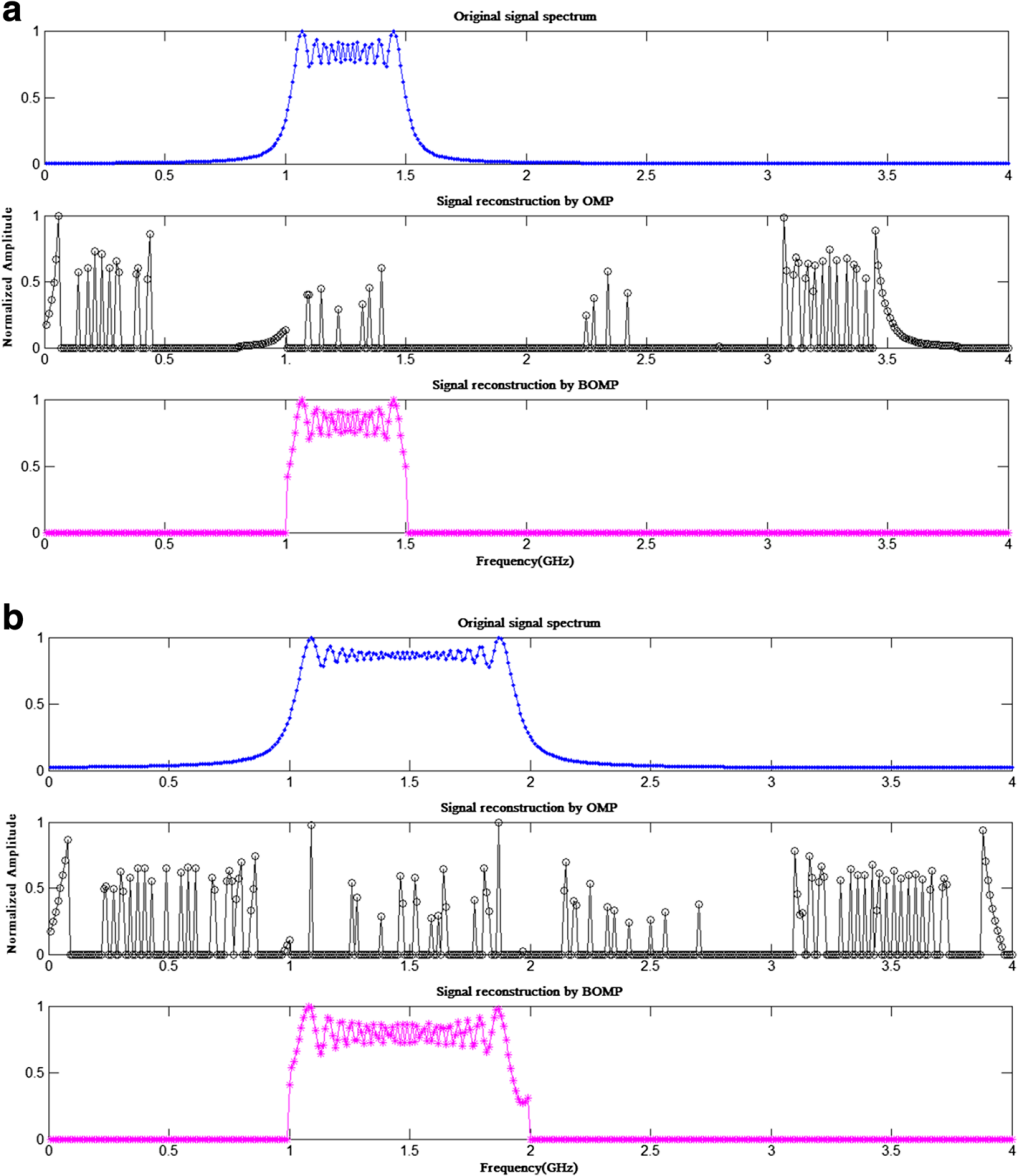
The broadband signal reconstruction by the CS algorithm

Figure 5 is the broadband signal reconstruction by using the BOMP algorithm based on the block-RIP analysis by comparing with the OMP reconstruction. Meanwhile, recovering of the spectrally sparse signals with single-frequency by using the OMP algorithm is applied, as shown in [1], and not to be repeated again here. The simulation settings are the same in Table 1, except the average sampling frequency is changed into 0.5 GHz to save the simulation time. The initial frequency of the broadband LFM signal shown in Fig. 5a is 1 GHz, the amplitude is 1, the phase is 0, and the bandwidth is 0.5 GHz with the pulse width of 0.1 μs. Then, the signal is 1-block sparse of size 100 and can be recovered successfully. This example illustrates that the measurement matrix of the NYFR satisfies 1-block-RIP.

Figure 5b shows the broadband signal reconstruction with the bandwidth of 1 GHz, which covered two Nyquist zones under 50 % cover rate of the receiver bandwidth. As we can see, the block CS analysis is more suitable for the NYFR, especially for the broadband signal receiving. It is implied that the modulated sampling schemes, as the architecture of the NYFR, may have advantages for data compression and transmission, which lies at the middle of the uniform and random sampling.

## Conclusions

This paper discusses the RIP and block-RIP analysis of the NYFR for recovering signals. The contribution of this work is to analyze the RIP of the NYFR as a phase-modulated sampling scheme based on the CS model. And, we get the conclusion that the NYFR suffers a poor RIP for the broadband signal, because the broadband signal does not satisfy the sparse condition, which will lead the conventional CS algorithms to be invalid. By deriving the Fourier spectrum of the broadband signal, which covered multiple Nyquist zones and received by the NYFR, the broadband signal can be regarded as the block-sparse signal. Then, the block CS algorithms can be applied for recovering the signal, and the block-RIP of the NYFR is demonstrated deterministically to show the reconstruction probability. Simulation verifies the correctness of the RIP and block-RIP theoretical findings of the NYFR. And, there is the broadband signal reconstruction by comparing the method of OMP and BOMP. Future research will aim to investigate the adaptive recognition algorithm of the partition blocks, including extending the signal to other sparse domain.

## Abbreviations

RF: Radio frequency
A2I: Analogy-to-information
ADC: Analog-to-digital converter
RIP: Restricted isometry property
block-RIP: Block restricted isometry property
OMP: Orthogonal matching pursuit
DFT: Discrete Fourier transform
NYFR: Nyquist folding receiver
CS: Compressive sensing
EW: Electronic warfare
RIC: Restricted isometry constant
block-RIC: Block restricted isometry constant
BOMP: Block orthogonal matching pursuit
IDFT: Inverse discrete Fourier transform
